# Evaluation of [^68^Ga]Ga-DOTA-AeK as a Potential Imaging Tool for PET Imaging of Cell Wall Synthesis in Bacterial Infections

**DOI:** 10.3390/ph17091150

**Published:** 2024-08-31

**Authors:** Palesa C. Koatale, Mick M. Welling, Sipho Mdanda, Amanda Mdlophane, John Takyi-Williams, Chrisna Durandt, Iman van den Bout, Frederik Cleeren, Mike M. Sathekge, Thomas Ebenhan

**Affiliations:** 1 Department of Nuclear Medicine, University of Pretoria, Pretoria 0001, South Africa; palesa.koatale@up.ac.za (P.C.K.); sipho.mdanda@sanumeri.co.za (S.M.); amanda.mdlophane@sanumeri.co.za (A.M.); mike.sathekge@up.ac.za (M.M.S.); 2 Nuclear Medicine Research Infrastructure (NuMeRI), Steve Biko Academic Hospital, Pretoria 0001, South Africa; 3 Department of Radiology, Interventional Molecular Imaging Laboratory, Leiden University Medical Center, 2333 ZA Leiden, The Netherlands; m.m.welling@lumc.nl; 4 Therapeutics Systems Research Laboratories (TSRL), Inc., Ann Arbor, MI 48109, USA; jtakyiwilliams@tsrlinc.com; 5 Department of Medical Immunology, Institute for Cellular and Molecular Medicine, University of Pretoria, Pretoria 0001, South Africa; chrisna.durandt@up.ac.za; 6 South African Medical Research Council Extramural Unit for Stem Cell Research and Therapy, University of Pretoria, Pretoria 0001, South Africa; 7 Department of Physiology, University of Pretoria, Pretoria 0001, South Africa; iman.vandenbout@up.ac.za; 8 Department of Pharmacy and Pharmacological Sciences, Radiopharmaceutical Research, KU Leuven, 3000 Leuven, Belgium; frederik.cleeren@kuleuven.be

**Keywords:** PET tracer, cell wall, synthesis, purification, quality control, bacterial infection, PET imaging

## Abstract

The ability of bacteria to recycle exogenous amino acid-based peptides and amino sugars for peptidoglycan biosynthesis was extensively investigated using optical imaging. In particular, fluorescent AeK–NBD was effectively utilized to study the peptidoglycan recycling pathway in Gram-negative bacteria. Based on these promising results, we were inspired to develop the radioactive AeK conjugate [^68^Ga]Ga-DOTA-AeK for the in vivo localization of bacterial infection using PET/CT. An easy-to-implement radiolabeling procedure for DOTA-AeK with [^68^Ga]GaCI_3_ followed by solid-phase purification was successfully established to obtain [^68^Ga]Ga-DOTA-AeK with a radiochemical purity of ≥95%. [^68^Ga]Ga-DOTA-AeK showed good stability over time with less protein binding under physiological conditions. The bacterial incorporation of [^68^Ga]Ga-DOTA-AeK and its fluorescent Aek-NBD analog were investigated in live and heat-killed *Escherichia coli* (*E. coli*) and *Staphylococcus aureus* (*S. aureus*). Unfortunately, no conclusive in vitro intracellular uptake of [^68^Ga]Ga-DOTA-AeK was observed for *E. coli* or *S. aureus* live and heat-killed bacterial strains (*p* > 0.05). In contrast, AeK-NBD showed significantly higher intracellular incorporation in live bacteria compared to the heat-killed control (*p* < 0.05). Preliminary biodistribution studies of [^68^Ga]Ga-DOTA-AeK in a dual-model of chronic infection and inflammation revealed limited localization at the infection site with non-specific accumulation in response to inflammatory markers. Finally, our study demonstrates proof that the intracellular incorporation of AeK is necessary for successful bacteria-specific imaging using PET/CT. Therefore, Ga-68 was not a suitable radioisotope for tracing the bacterial uptake of AeK tripeptide, as it required chelation with a bulky metal chelator such as DOTA, which may have limited its active membrane transportation. An alternative for optimization is to explore diverse chemical structures of AeK that would allow for radiolabeling with ^18^F or ^11^C.

## 1. Introduction

Bacterial infections remain a significant health concern, in part due to the growing incidence of antibacterial resistance. Accurate diagnosis of bacterial infections is crucial to avoid unnecessary antibiotic use, and implementing effective antibiotic stewardship requires rapid diagnostic tests [1]. However, the traditional approach to diagnosing infections requires sampling bodily fluids or performing an invasive biopsy followed by microbiological culturing or molecular typing, which often requires several days to weeks to obtain results [2,3]. To address these limitations, morphological imaging techniques, including ultrasound, computed tomography (CT), and magnetic resonance imaging (MRI), have been used for real-time, non-invasive diagnosis. However, early infections and infections with dormant bacteria often remain undetectable [4]. As a result, a dual-nuclear imaging modality such as positron emission tomography/ computed tomography (PET/CT) is considered a good alternative due to its ability to non-invasively assess anatomical and physiological abnormalities, especially in the early stages of infection or for deeply seated infections, where sampling is challenging.

PET/CT can be used efficiently in monitoring treatment response and relapse [5,6]. For infection detection and imaging, current nuclear medicine techniques rely on direct or indirect targeting of infected sites using [^67^Ga]Ga-Citrate ([^99m^Tc]Tc/[^111^In]-labeled leucocytes) or via active glucose metabolism using 2-deoxy-2[^18^F]fluoro-D-glucose ([^18^F]-FDG). Unfortunately, these radiotracers fail to discriminate infection from inflammation and often suffer from poor resolution [7,8,9]. Thus, the current clinical radiotracers have limited diagnostic power in differentiating infections from other inflammatory processes. Unlike eukaryotic cells, bacteria possess a complex and unique biological cell machinery that offers multiple opportunities for pathogen-specific targeting. Therefore, improvements in the diagnostic strategy require the development of tailored radiotracers depending on bacteria-specific features that allow for rapid and specific localization of infectious foci that may be selective for the type of bacteria involved in an infection [10,11]. With this shift in focus, several potential candidates targeting bacterial biochemical processes have emerged, including antibiotics ([^99m^Tc]Tc-/[^18^F]F-Ciprofloxacin), antimicrobial peptides ([^99m^Tc]Tc/[^68^Ga]Ga-UBI_29–41_), metabolites ([^18^F]F-D-sorbitol), antibodies ([^89^Zr]Zr-human monoclonal antibody 1D9), bacteriophages ([^99m^Tc]Tc-M13 phages) and oligomers (e.g., [^99m^Tc]Tc-MORF oligomer) [12,13].

**Figure 1:**
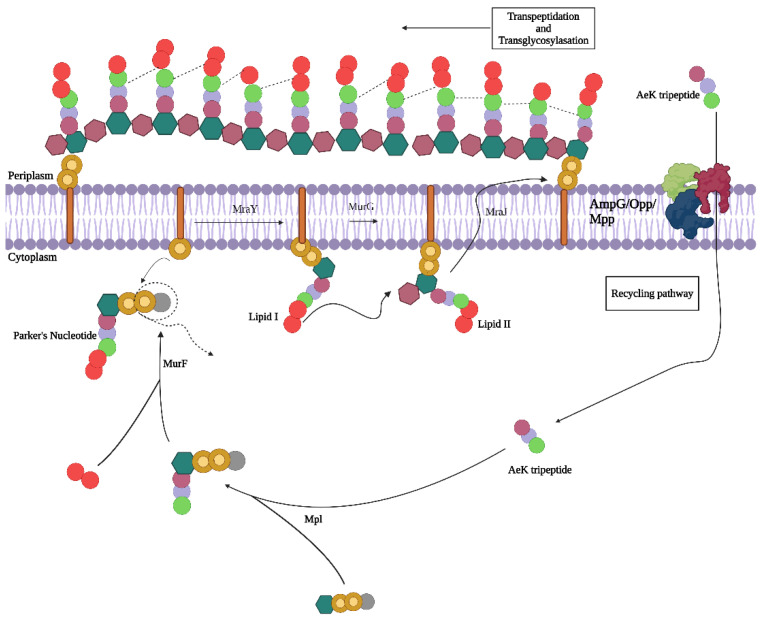
Proposed peptidoglycan recycling targeting pathway of AeK tripetide. Adapted with permission from ref [19], published in 2024 under a Creative Commons Attribution 4.0 International License. Created with BioRender.com.

Of particular interest is the bacterial cell wall, which is of use in classifying bacteria (Gram-negative versus Gram-positive) based on the complexity and heterogeneity of the peptidoglycan (PG) building blocks [14]. Evident in all bacteria, PG forms an integral net-like structure of the cell wall, composed of alternating N-acetylglucosamine and N-acetylmuramic acid amino sugars cross-linked by oligopeptides. PG biosynthesis and remodeling involve a well-understood, dynamically regulated mechanism [15]. During cell proliferation, the PG polymer is enzymatically digested into L-Ala-γ-D-Glu-meso-A2pm (or L-Lys)-D-Ala tetrapeptide and L-Ala-γ-D-Glu-meso-A2pm (or L-Lys) tripeptides, and then enters the PG recycling pathway via oligopeptide permease (Opp) membrane protein transporters found in both *Escherichia coli* (*E. coli*) and *Staphylococcus aureus* (*S. aureus*) [16]. Once in the intracellular space, the amino acids chains (tri- and tetrapeptides) are covalently linked to amino sugars by murein peptide ligase (Mpl), resulting in the formation of uridine diphosphate-N-acetylmuramic acid (UDP-MurNAc)-tripeptide/tetrapeptides, a process which is integral for the reintegration of the peptides into the bacterial cell wall. These muropeptides are further processed and incorporated into a newly formed PG layer during cell proliferation (Figure 1) [15,17,18].

**Figure 2:**
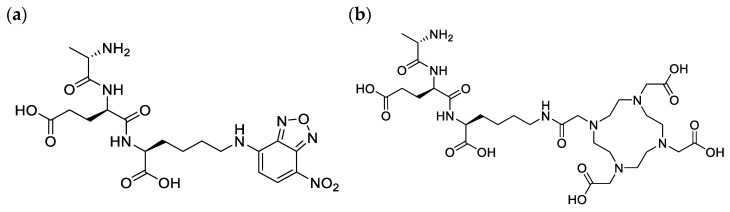
Chemical structure of (**a**) AeK–NBD and (**b**) AeK–DOTA.

We recently studied PG targeting molecules and reviewed their potential as future agents for PET imaging of infection [19]. Various studies have demonstrated the ability of bacteria to utilize synthetic D-amino acids and tripeptides for the reconstruction of the PG, and that extends to their fluorescence and radioactive analogs [20,21,22,23]. For example, in a study by Goodell [24], the radiolabeled tripeptide L-Ala-γ-D-Glu-[^3^H]A2pm was effectively utilized by *E. coli* in the PG recycling pathway without tracer degradation. Interestingly, similar results were reported in another study using the fluorescent tripeptide L-Ala-γ-D-Glu-L-Lys-N-7-nitro-2,1,3-benzoxadiazol-4-yl (AeK–NBD; Figure 2a), in which the conjugation of diaminopimelic acid to the epsilon amino group of lysine did not compromise the bacterial uptake [25].

In recent years, we have seen an evolvement in the synergistic approach to using translate fluorescent probes for targeting the bacterial cell wall, primarily limited to in vitro into infection imaging radiotracers for the advancement of target-specific diagnostic tools, which is a critical need in the medical field [14]. In the current study, we intended to replace the fluorescent entity NBD with the bifunctional chelator 4,7,10 tetraaza-cyclododecane-N,N′,N″,N‴-tetraacetic acid (DOTA), thereby forming L-Ala-γ-D-Glu-L-Lys-DOTA (AeK–DOTA, Figure 2b) to allow for radiolabeling with the radioisotope Gallium-68 (^68^Ga) for prospective bacterial imaging using PET/CT. A ^68^Ga-labeling method for AeK-DOTA was developed, followed by a tailored purification procedure for [^68^Ga]Ga-DOTA-AeK to enable initial radiopharmacological and physicochemical characterization (lipophilicity, protein binding, and plasma stability). Subsequently, a preliminary animal study was performed using [^68^Ga]Ga-DOTA-AeK-PET/CT for pharmacokinetics and biodistribution investigations to study its suitability for PET imaging.

## 2. Results and Discussion

### 2.1. Development of ^68^Ga Radiolabeling Method for DOTA-AeK

**Figure A1:**
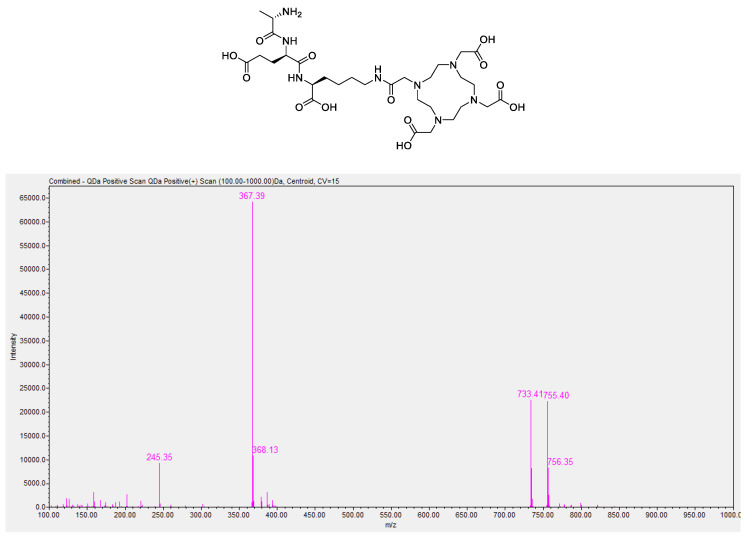
LC/MS spectrum of DOTA-AeK showing *m*/*z* ratios of 733.4 ([M+H]^+^), 734.4 ([M+H]^2+^), 755.40 ([M+H+Na]^+^), 756.35 ([M+H+Na]^2+^), 367.39 (½[M+H]^1+^), and 368.13 (½[M+H]^2^).

**Figure A2:**
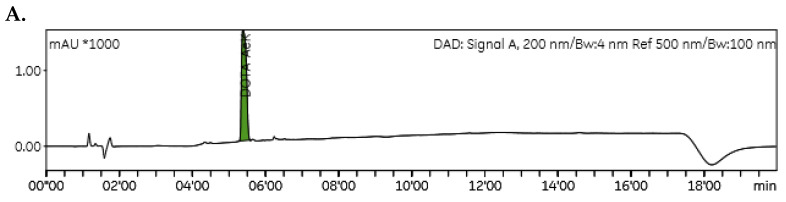

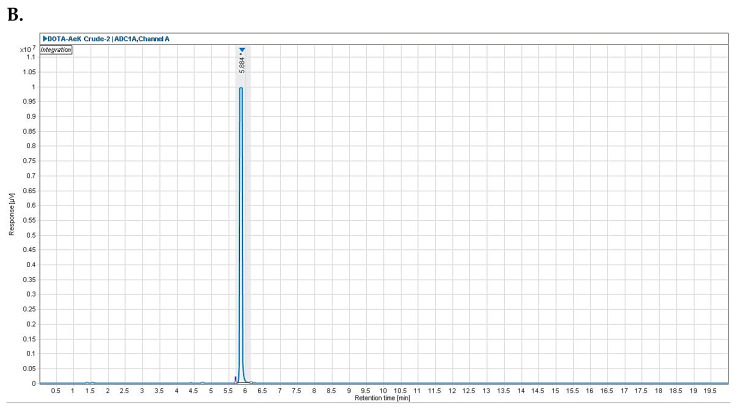
Representative analytical HPLC chromatogram of (**A**) DOTA-AeK with a retention time of 5.2 min and (**B**) crude *[^68^Ga]Ga-DOTA-AeK with a retention time 5.9 min.

Aside from various peptides with longer amino acid sequences, we previously developed kit-based radiosynthesis for the pentapeptide NOTA-cyclo-Arg-Gly-Asp-d-Tyr-Lys (NOTA-RGD) [26]; this confirmed the safe use of sodium acetate as a buffering agent, which was subsequently used in this study. Furthermore, original results concerning the radiosynthesis of DOTA-AeK (732.79 g/mol, Figure A1 and Figure A2A) with Ga-68 are reported below.

**Figure A3:**
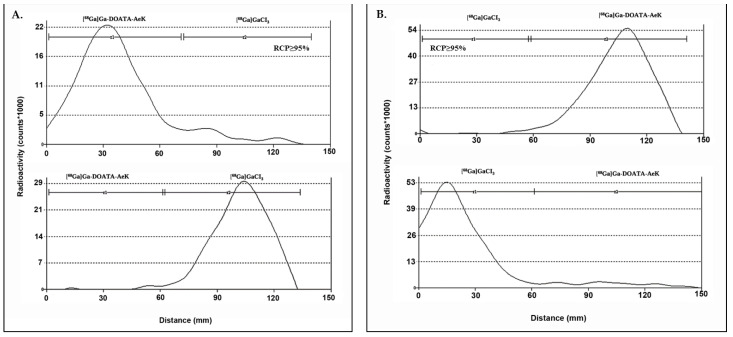
ITLC chromatogram of (**A**) [^68^Ga]Ga-DOTA-AeK (top) and [^68^Ga]GaCl_3_ (pH 4) (bottom) using (**A**) mobile phase A: 0.1 M citric acid (pH 5.0); (**B**) mobile phase B: 1 M Ammonium acetate: methanol (1:1).

Firstly, to ensure accurate analysis of radiosynthesis, the validity of radioanalytical methods for [^68^Ga]Ga-DOTA-AeK quality control were tested. Both HPLC and ITLC methods were assessed for their ability to identify [^68^Ga]Ga-DOTA-AeK and were found to accurately measure the compound. Thus, both methods can be used as radioanalytical quality control measurements for crude and purified [^68^Ga]Ga-DOTA-AeK samples. The required labeling efficiency and product purity was confirmed via radio-HPLC (Figure A2B) and the ITLC method (Figure A3A,B and Table A1).

#### 2.1.1. Effects of Radiolabeling Conditions on Ga-68-Complexation of DOTA-AeK

DOTA was selected as a chelator, along with a 95 °C incubation temperature and sodium acetate as the buffering agent (pH capacity range 3.7–5.6), with eluate fractionation as the preferred generator elution technique based on previous experience and similar outcomes from the radiolabeling of different research peptides [27,28,29].

Therefore, crucial changes to vector concentration, eluate acidity, and incubation time were investigated to determine the optimal radiolabeling parameters. These parameters were set at ≥95% RCP.

#### 2.1.2. Ga-68-Eluate Acidity and Vector Concentration (DOTA-AeK)

**Figure 3:**
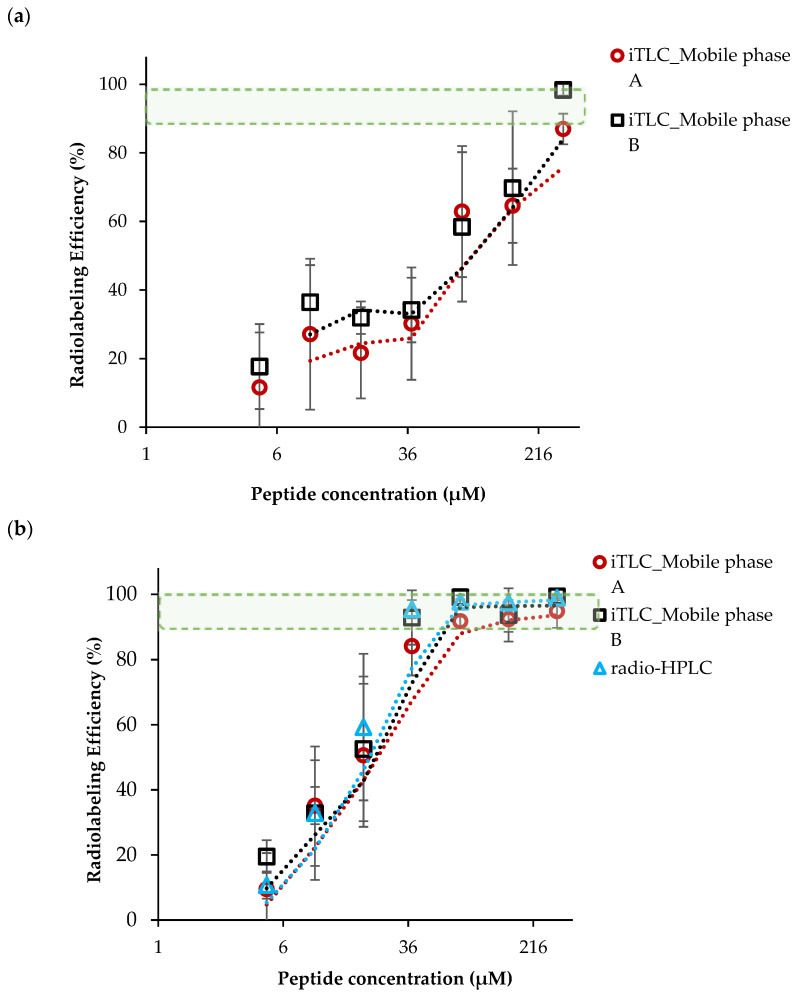
The influence of peptide concentration and pH on radiolabeling efficiency performed at (**a**) pH 3 and (**b**) pH 4 (95 °C, 15 min). The results are expressed as mean ± SD (n = 3) with fitted lines indicated. The green-shaded area indicates the desired level of radiolabeling efficiency.

A serial radiolabeling strategy was carried out to investigate the influence of pH (3 and 4) and vector concentration on the complexation of [^68^Ga]GaCI_3_ and DOTA-AeK. Figure 3a,b show that radiolabeling efficiency is directly proportional to the vector concentration. In addition, the data demonstrate the critical role the pH plays in coordinating the chemistry of [^68^Ga]GaCl_3_ with DOTA. At pH 3, a radiolabeling efficiency of at least ≥85.9 ± 2.3% was observed at the highest peptide concentration of 303 µM. In contrast, the labeling efficiency was significantly improved at pH 4, with the highest RCY of ≥95% obtained from a 76 µM peptide concentration. This difference in efficiency may have been a result of carboxylic acid group protonation and the formation of hydrolyzed ^68^Ga^3+^ colloidal species [^68^Ga(H_2_O)_6_]^3+^ observed at pH ≤ 3.5, resulting in lower RCY. At pH 4, the carboxylic acid group were partially deprotonated, forming an ionic bond with ^68^Ga^3+^ and optimum RCY at a low peptide concentration [30,31,32,33].

#### 2.1.3. Optimization of Incubation Parameters

**Figure 4:**
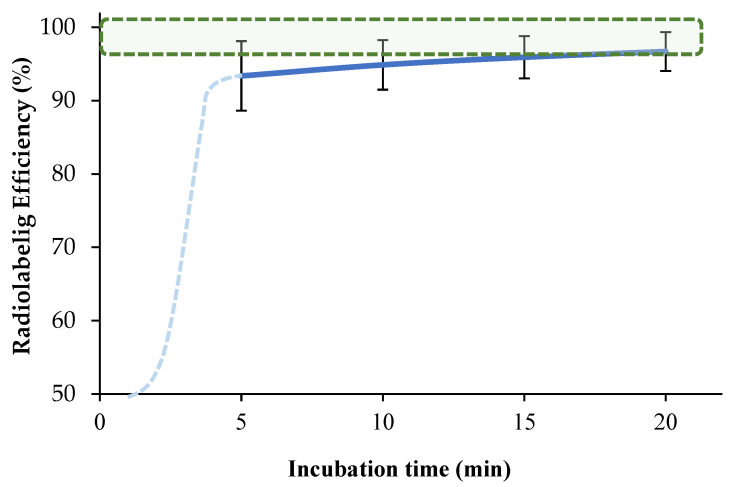
The effect of incubation time on the complexation of [^68^Ga]GaCI_3_ with DOTA-AeK (76 µM; pH 4; 95 °C) up to 20 min using radio-HPLC. The results are represented as mean ± SD (n = 3). The green-shaded area shows the required radiolabeling efficiency, >95%.

To investigate the effect of incubation time on the labeling efficiency, 76 µM [^68^Ga]Ga-DOTA-AeK was prepared via incubation for different durations (5–20 min). Our findings show an optimum radiolabeling efficiency of ≥95% between 10 and 20 min at 95 °C for [^68^Ga]Ga-DOTA-AeK (Figure 4, green areal). This is mainly due to the fairly slow reaction kinetics of DOTA (the dotted light blue line was added for kinetics that were estimated early-on), requiring a higher heating temperature (60–100 °C) for extended reaction times (range 10–60 min) for radiometal complexation to occur [34,35].

To maintain optimum labeling efficiency and %RCP, the following parameters were adopted for all routine radiolabeling during [^68^Ga]Ga-DOTA-AeK characterization and its preclinical assessment: 76 µM DOTA-AeK mixed with Ga-68 eluate buffered with sodium acetate to an acidity of pH 4.0 followed by 15 min incubation at 95 °C.

### 2.2. Development of a Purification Method of [^68^Ga]Ga-DOTA-AeK

Solid-phase extraction (SPE) is often suggested for improving the radiochemical purity (RCP) of a radiotracer via the removal of impurities, such as uncomplexed-^68^Ga, colloidal-^68^Ga, traces of Germanium-68 (^68^Ge) and co-eluted metals [36,37]. Here, we tested the product purification procedure for radiolabeled [^68^Ga]Ga-DOTA-AeK using the different strategies tested, which are listed in Table A2.

Firstly, the cartridge retention of [^68^Ga]Ga-DOTA-AeK by different cartridges was investigated (Table A2). The C18 cartridge showed a maximum retention capacity of 84.9 ± 10.6% of the total amount of radiotracer radioactivity applied, while the lowest retention capacities were observed with the HBL and Stata X cartridges. Since C18 showed the highest [^68^Ga]Ga-DOTA-AeK retention, it was chosen for further optimization. It was hypothesized that the retention capacity of C18 cartridges could be negatively affected by sample overload, with 15.2 ± 10.6% of the radiotracer detected in the flow-through when using radio-HPLC (Table A2); therefore, two C18 cartridges were combined (290 mg sorbent mass) for purification to test this theory. Based on our results, the 290 mg sorbent C18 cartridge improved the retention capacity, allowing it to reach ≥98% (Table A2). Based on these observations, we conclude that the combined cartridge capacity significantly improved the retention capacity. This finding agrees with that of a study by Alhankawi et al. [38], which reported the improved retention capacity of hydrophilic peptides using C18-C18 loaded with a larger mass of absorbent.

The second optimization procedure concerned the critical effects of wash/rinse solution and volume. and compared the consistencies of purification performance for recovery efficiency and radiotracer purity (Table 1). Although it was possible to retain [^68^Ga]Ga-DOTA-AeK on a non-polar C18 absorbent, removing impurities using 1 mL of an aqueous solution resulted in cartridge retention loss with 85.8 ± 6.9% activity reported post-wash. This was mainly due to the hydrophilic properties of [^68^Ga]Ga-DOTA-AeK. Therefore, reducing the washing solution volume (0.4 mL) minimized the radiotracer loss to a reported 94.9 ± 6.2% retained radioactivity (Table 1).

Finally, efficient recovery of [^68^Ga]Ga-DOTA-AeK from the cartridge was evaluated using different ethanol concentrations (Table A2). Due to the hydrophilic properties of [^68^Ga]Ga-DOTA-AeK, 5% *v*/*v* ethanol solution was sufficient to recover the compound, resulting in a high RCP of ≥95% and an excellent recovery of 83.5 ± 7.6%.

Based on these results, purification using the 290 mg C18 cartridge, 0.4 mL 1× PBS wash solution, and 5% *v*/*v* EtOH (in PBS pH 7.4) for elution was adopted as a standard SPE purification method for [^68^Ga]Ga-DOTA-AeK.

### 2.3. Log P Determination for [68Ga]Ga-DOTA-AeK

The determination of the radiotracer’s hydrophobicity is a critical part of development as it critically affects the biodistribution of the radiotracer [39]. The hydrophobicity of [^68^Ga]Ga-DOTA-AeK was investigated using the octanol/water partition procedure. Based on the results, 92.3 ± 0.04% of the tracer was recovered within the aqueous phase with a calculated partition coefficient (Log P) of −1.08 ± 0.00 (n = 3). The results indicate that [^68^Ga]Ga-DOTA-AeK shows a high level of hydrophilicity.

### 2.4. Serum Protein Binding of [^68^Ga]Ga-DOTA-AeK and Proteolytic Stability

**Figure 5:**
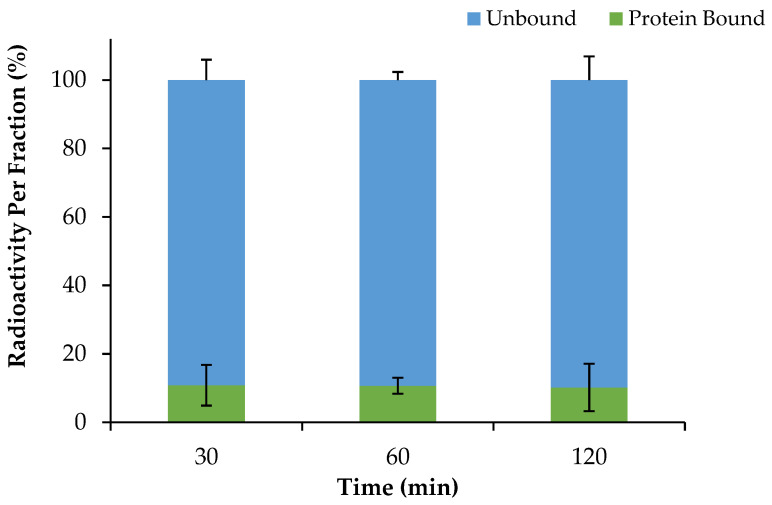
Plasma protein binding of [^68^Ga]Ga-DOTA-AeK (37 °C; over 120 min). The results are represented as mean ± SD (n = 3).

The plasma protein binding properties of [^68^Ga]Ga-DOTA-AeK were investigated using rapid protein ultrafiltration and separation at different time points. Based on the results (Figure 5), [^68^Ga]Ga-DOTA-AeK demonstrated low protein binding properties with an approximately 10.18 ± 6.9% protein-bound fraction, recorded up to 120 min with no significant difference observed between 30 and 120 min. This observation is mainly attributed to the hydrophilic nature of the radiotracer, as previous studies have demonstrated a positive correlation between hydrophobicity and plasma protein binding [38,40].

**Figure 6:**
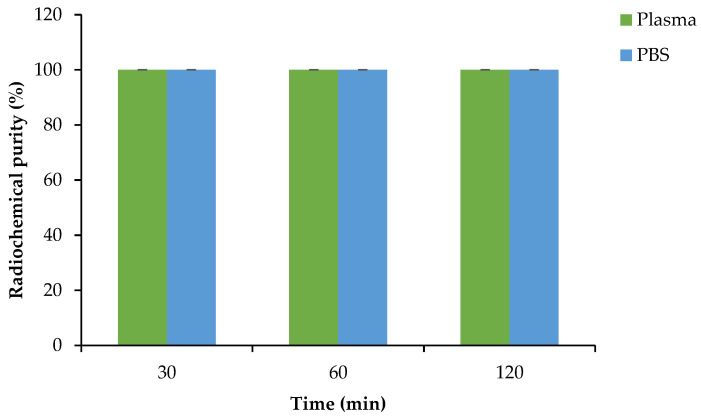
Plasma and PBS stability of [^68^Ga]Ga-DOTA-AeK at 37 °C. The results are represented as mean ± SD (n = 3).

After centrifugal filtration, the unbound radioactivity found in the liquid fraction in the collection tubes was further tested for plasma and PBS stability and analyzed via radio-HPLC. [^68^Ga]Ga-DOTA-AeK showed no degradation in plasma and PBS (control) over an extended incubation time, thereby maintaining the desired RCP of ≥95% (Figure 6).

### 2.5. Formulation Stability

**Figure 7:**
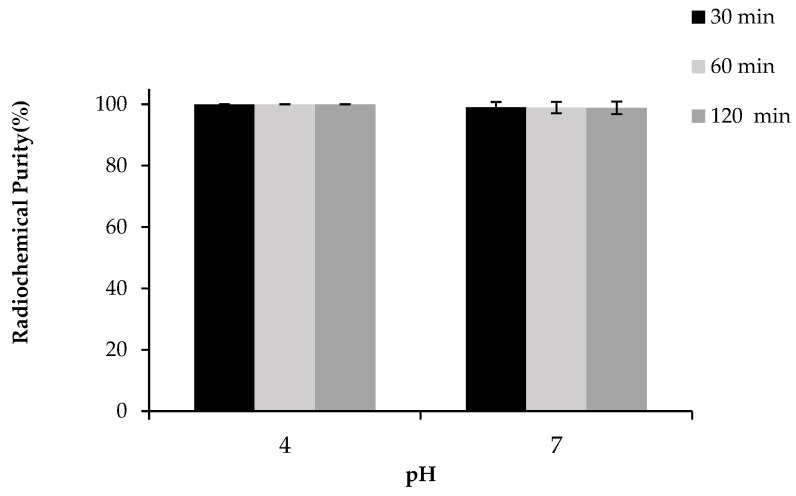
Stability of purified [^68^Ga]Ga-DOTA-AeK formulation (pH 4.0 and 7.0; room temperature; up to 120 min) analyzed via radio-HPLC (re-occurrence of free Ga-68 species). The results are represented as mean ± SD (n = 3).

The stability of [^68^Ga]Ga-DOTA-AeK was tested in different product formulations (pH 4.0 and 7.0) post-purification at room temperature. As displayed in Figure 7, the post-purification eluate (pH 4.0) and neutralized formulation (pH 7.0) remained stable up to 120 min with an RCP of 100 ± 0% and 98.8 ± 2.0%, respectively. In neutral and slightly basic conditions, deprotonation of donor atoms (e.g., nitrogen) might occur, potentially increasing the metal-binding capacity. However, if the solution becomes too basic, the metal ion may hydrolyze, forming metal hydroxides, which reduces the stability and purity of the radiotracer, with a non-specific distribution likely to be seen in the liver, spleen, and bone [41,42]. Based on the results, we achieved a <10% *v*/*v* EtOH formulation with the RCP of ≥95% required for preclinical investigations.

### 2.6. Bacterial Cell Uptake of [^68^Ga]Ga-DOTA-AeK

**Figure 8:**
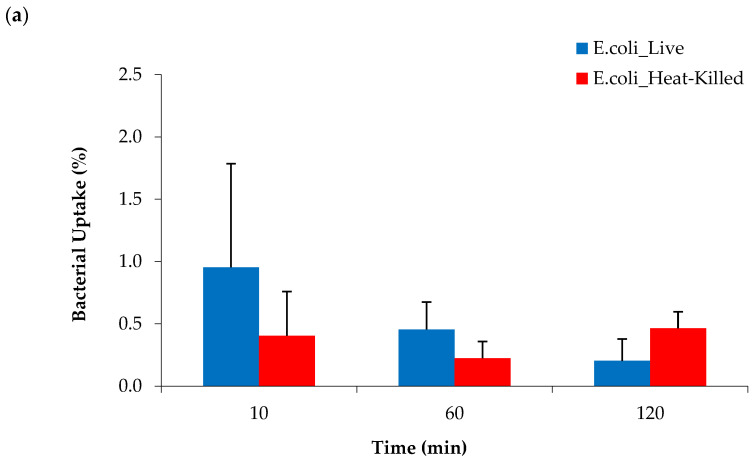

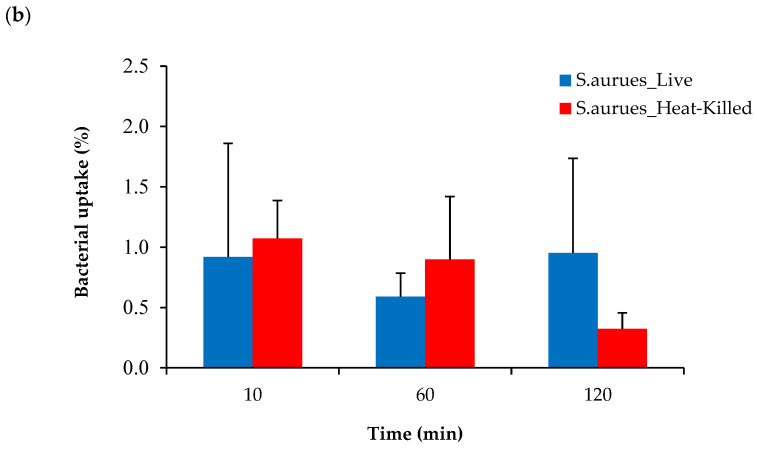
Cellular uptake of [^68^Ga]Ga-DOTA-AeK in live and heat-killed bacterial cultures of (**a**) *E. coli* and (**b**) *S. aureus* incubated at 37 °C for up to 120 min (n = 3). Unpaired Student’s *t*-tests were performed for comparison. *p* < 0.05 values were considered statistically significant. All measured values were not significant.

To test if bacteria were able utilize [^68^Ga]Ga-DOTA-AeK, in vitro studies were performed by incubating live and heat-killed bacterial cultures of *E. coli* and *S. aureus* with the tracer for different time periods. No significant increase in radioactivity above the heat-killed bacterial cells was measured at any time point up to 120 min with ~98% of the radiotracer detected in the medium (Figure 8a,b). Similar to our results, a lack of bacterial uptake and internalization was previously reported for ^3^H-labeled disaccharide-(L-Ala-D-Glu-meso-A2pm) tripeptide (800 g/mol), an analogue of AeK, while the same study demonstrated successful in vitro integration of L-Ala-γ-D-Glu-[^3^H]A2pm into the PG layer of *E. coli* [24]. Thus, in vitro results of our study suggest the unsuccessful integration of [^68^Ga]Ga-DOTA-AeK.

**Figure 9:**
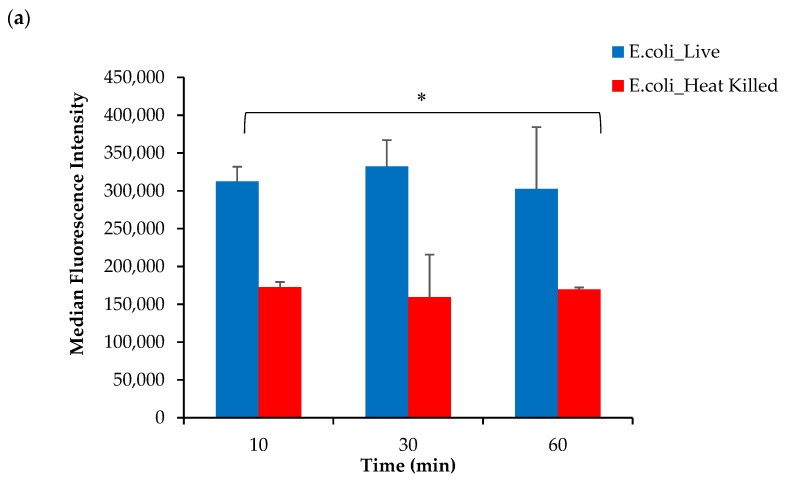

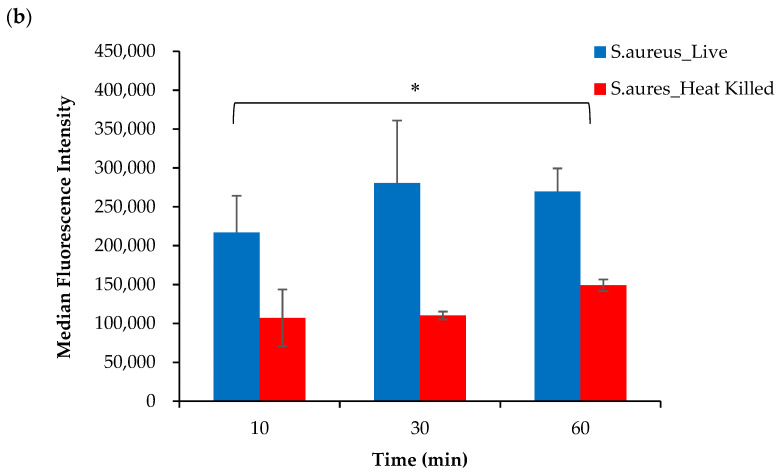
Flow cytometry analysis of live and heat-killed (**a**) *E. coli* and (**b**) *S. aureus* cell cultures incubated with AeK-NBD for up to 60 min (counterstained with Vybrant DyeCycle). Results are represented as mean and SD (n = 2 for each parameter). Unpaired Student’s *t*-tests were performed for comparison, with *p* values < 0.05 (*) considered statistically significant.

**Figure A4:**
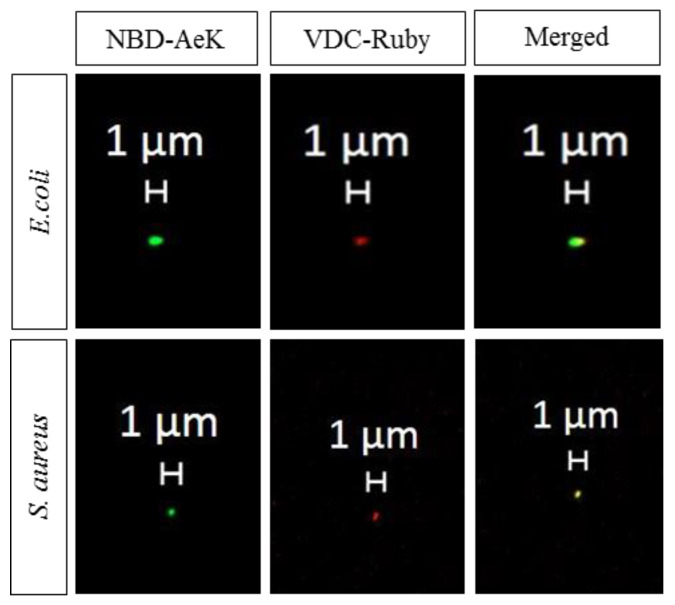
Representative confocal images of live *E. coli* and *S. aureus* cells labeled with NBD-AeK (green) and counterstained with the DNA stain VDC Ruby dye (red).

Based on these findings, it was hypothesized that the conjugation of DOTA to the AeK tripeptide interfered with the targeting properties of [^68^Ga]Ga-DOTA-AeK. To justify this interpretation, the in vitro incorporation of the initially studied fluorescent analogue of AeK (AeK-NBD) was measured in live and heat-killed *E. coli* and *S. aureus* cells using flow cytometry and confocal imaging. The flow cytometry analysis demonstrated (Figure 9a,b) high fluorescence intensity in both live *E. coli* and *S. aureus* cells, which was significantly decreased in heat-killed cell cultures (*p* < 0.05). Furthermore, as demonstrated in our experiments and a previous study [25], the bacterial incorporation of AeK-NBD was observed at 10 min, indicating that uptake occurred immediately after adding the tracer to the bacterial cultures (Figure 9a,b). This observation was supported by confocal imaging (Figure A4).

Therefore, it is plausible from previous studies that PG-targeting tripeptides containing either diaminopimelic acid (A2pm) or lysine can be taken up by bacteria, which was evident with [^3^H]L-Ala-γ-D-Glu-meso-A2pm and AeK-NBD [24,25]. Interestingly, contrary to previous findings in the literature, we demonstrated bacterial uptake of AeK-NBD not only in *E. coli* but also in *S. aureus* cultures, which was not tested for accuracy and specificity. However, our data suggest that although DOTA-AeK (732.79 g/mol) and AeK-NBD (509.48 g/mol) have the same targeting entity (AeK), the imaging vector can interfere with the metabolic integration of AeK into the PG macrostructure. There is some evidence supporting the assumption of a molecular weight cut off, with a ≤600 g/mol requirement for active membrane transportation, as seen with other PG targeting molecules [43]. We may therefore attribute the lack of bacterial uptake seen with [^68^Ga]Ga-DOTA-AeK to its unfitting molecular size preventing active transportation or influx across the cellular membrane, and crucially hindering the intracellular enzymatic metabolism necessary for incorporating [^68^Ga]Ga-DOTA-AeK into the PG.

### 2.7. Exploratory Biodistribution of [^68^Ga]Ga-DOTA-AeK

#### PET/CT Imaging and Ex Vivo Biodistribution

**Figure 10:**
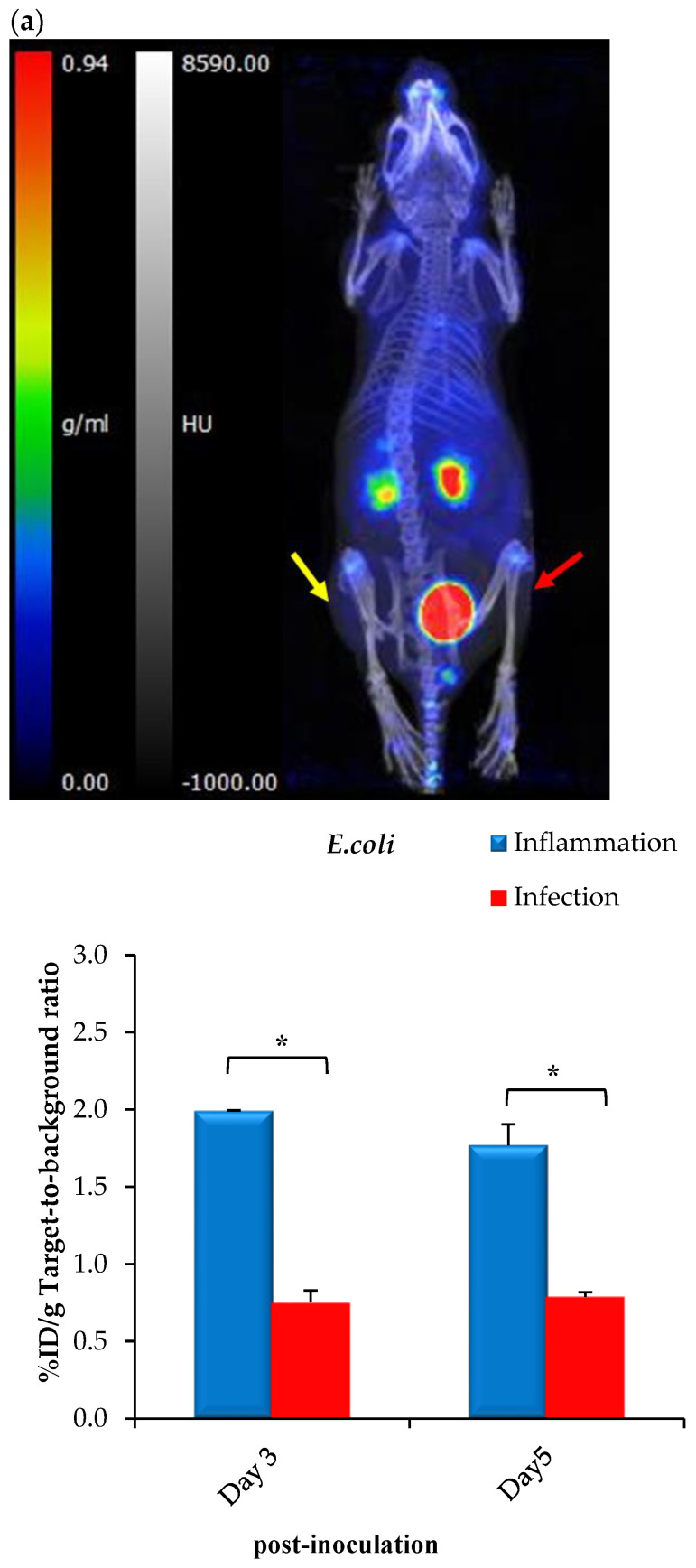

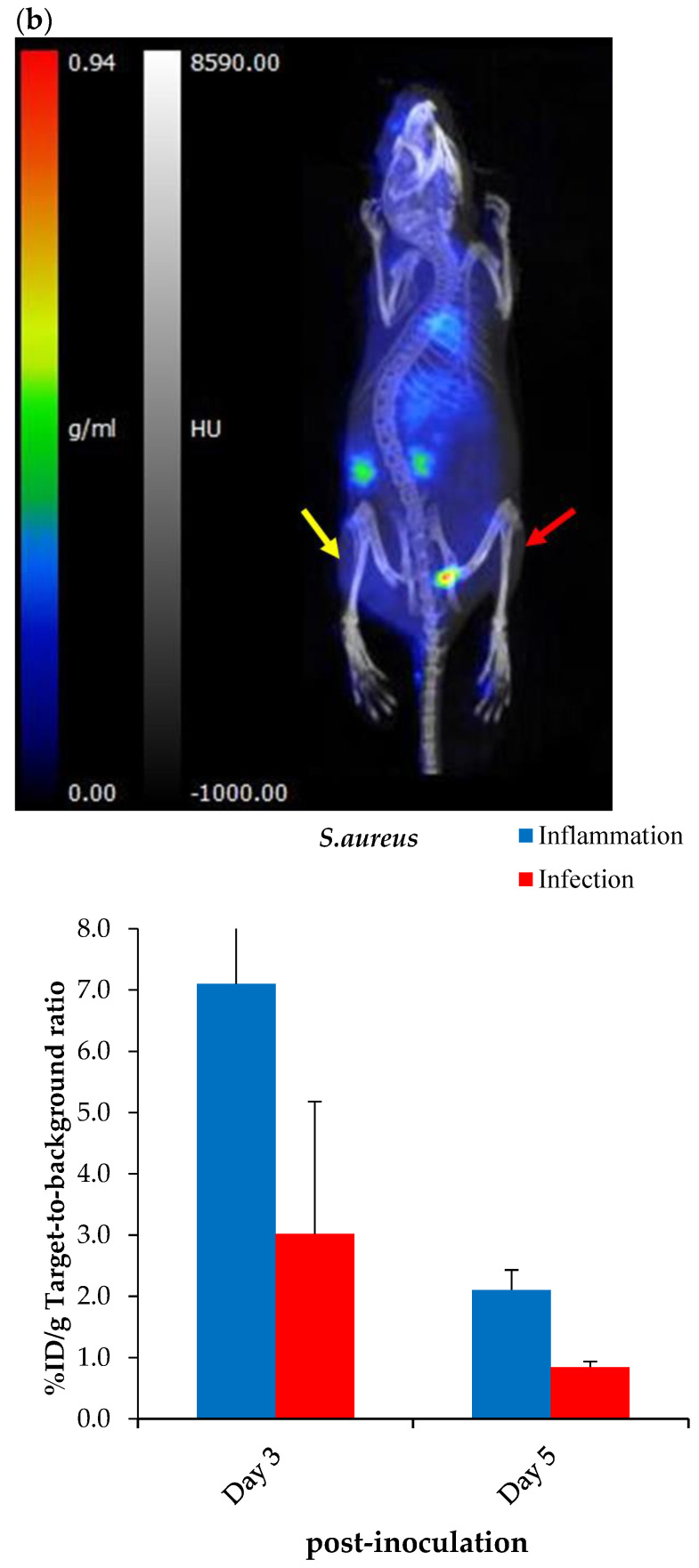
Representative PET/CT images and corresponding ex vivo biodistribution of [^68^Ga]Ga-DOTA-AeK after 60 min of intravenous injection in (**a**) *E. coli* and (**b**) *S. aureus* model of infection (red arrow) and inflammation (yellow arrow) at 3 and 5 days post-inoculation. Unpaired Student’s *t*-tests were performed for comparison, with *p* values < 0.05 (*) considered statistically significant.

Despite the lack of bacterial incorporation of [^68^Ga]Ga-DOTA-AeK in this study, the intracellular uptake observed earlier with AeK-NBD encouraged us to further characterize AeK in an in vivo setting, including PET imaging of infected animals. Therefore, preliminary in vivo biodistribution studies of [^68^Ga]Ga-DOTA-AeK were performed to understand the pharmacokinetics of AeK using *E. coli*- or *S. aureus*-bearing mice also suffering from sterile muscular inflammation. Results from qualitative PET/CT image analysis and ex vivo tissue counting suggest that [^68^Ga]Ga-DOTA-AeK showed no significant uptake in the infectious foci (tested at Day 3 and Day 5 post-inoculation of either *E. coli* or *S. aureus*). In contrast, the radiotracer was more pronounced in the sterile inflammation site in both cohorts (Figure 10a,b).

**Figure A5:**
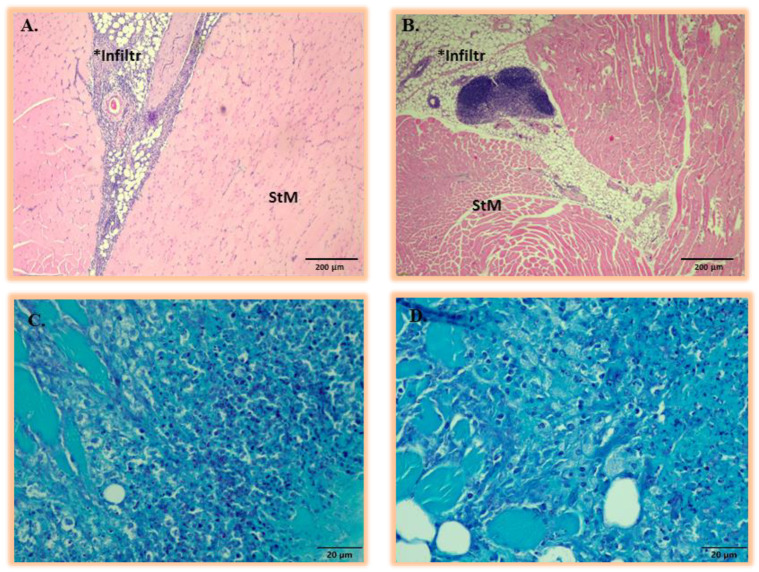
Representative histopathologic analysis of thigh muscular tissue at Day 3 and 5 post-inoculations with either (**A**) *E. coli* or (**B**) *S. aureus*. All the lesions induced by *E. coli* or *S. aureus* were identical and presented with moderate infiltration of neutrophils, macrophages, and lymphocytes (areas marked Infiltr *) within the adipose and striated muscle (StM). The corresponding Gram-stained sections of (**C**) *E. coli* or (**D**) *S. aureus* post-inoculation in thigh muscular tissue at Days 3 and 5 are shown with no visualization of representative bacterial strains.

However, the results should be interpreted with caution due to limitations in the experimental model, since active infections could not be confirmed (Figure A5A–D) and the imaging protocol was carried out later than at the standard 24 hrs post-inoculation. A critical reason is that after 3 or 5 days, most of the bacteria in the infected tissue were phagocytosed by host macrophages or otherwise had been encapsulated or included in a biofilm, therefore possibly being inaccessible for the radiotracer [44].

The ex vivo biodistribution data correlated with the findings from PET/CT imaging; see Figure 10a,b. Unlike other PG-targeting radiotracers [9,45,46,47], [^68^Ga]Ga-DOTA-AeK showed the minimum signal in background organs. Interestingly, significantly increased uptake was observed in the heart, liver and lungs of the *S. aureus* group at Day 5 post-inoculation and this is most likely to have been due to physiological changes induced from the initial infection and/or inflammation (Table A3). Despite traces of the radiotracer being detected in the liver, hepatobiliary excretion was excluded, with rapid clearance mainly via renal excretion. This can be explained by the hydrophilic properties of [^68^Ga]Ga-DOTA-AeK under physiological conditions. In addition, the in vivo stability of [^68^Ga]Ga-DOTA-AeK was confirmed as indicated, with very limited uptake of radioactivity in the bone (≤2%D/g) (Table A3).

## 3. Materials and Methods

Information on materials, biochemicals and selected equipment is referred to in Appendix A.

### 3.1. Testing Radioanalytical Methods for [^68^Ga]Ga-DOTA-AeK Quality Control

General equipment setting and processes (e.g., known stationary- and mobile-phase materials) for instant thin-layer chromatography (ITLC), high-performance liquid chromatography (HPLC) and liquid chromatography–mass spectrometry (LC/MS) were adopted from a previously published paper [48].

The purified DOTA-AeK was characterized via HPLC and LC/MS. The purity of DOTA-AeK was determined via a Waters Acquity LC/MS system equipped with a Waters XBridge BEH C18 column (3.5 µm, 100 mm × 3 mm, 130 Å) at a flow rate of 5 mL/min using a gradient of acetonitrile with 0.1% formic acid (5–95% over 10 min) and H_2_O with 0.1% formic acid. The HPLC method was first tested for its capability to accurately identify [^68^Ga]Ga-DOTA-AeK and separate it from various other radiolabeled by-products. An Agilent 1290 Infinity II HPLC apparatus equipped with an Agilent Infinity Lab Poroshell 120 EC-C18 (3.0 × 150 mm, 2.7 µm) column and a diode array UV detector (set at 220 nm) alongside a Gabi γ-HPLC flow detector (Elysia-raytest, Straubenhardt, Germany) were used for analysis. The method setup included a 20 min-long A/B gradient mobile phase protocol (0.5 mL/min flow rate; A: H_2_O, 0.1% TFA, B: acetonitrile, 0.1% TFA) with the elution set as follows: 0–1 min: 97% A; 1–10 min: decrease 97% A to 72% A; 10–15 min 72% A; 15–20 min: 97% A.

The ITLC method was first tested for its selectivity to determine the radiolabeling efficiency and radiochemical purity (RCP) in crude or purified [^68^Ga]Ga-DOTA-AeK product samples. Retention factors (R*f*) were determined to calculate the method resolution (R*s*). Peak identification was performed via baseline-gated “area-under-the-curve” analysis.

### 3.2. Development of a Radiosynthesis Method for [^68^Ga]Ga-DOTA-AeK

Optimal conditions for radiolabeling were first determined. For this purpose, a strategy described by Mokaleng et al. [49] was adopted to facilitate the general radiolabeling principle for DOTA-AeK by investigating parameters such as the influence of pH, compound concentration and incubation time on the labeling efficiency. First, the [^68^Ga]GaCl_3_ was eluted with 0.6 M HCI via generator eluate fractionation, and sodium acetate trihydrate solution (2.5M; pH 8.5) was used as the buffering agent. To investigate the effect of pH and the concentration parameters on the labeling yield, 2-fold serial dilutions of 25 µL of DOTA-AeK (0–137 µM) were prepared from stock solutions of DOTA-AeK (50 µg, 68.2 nmol) followed by the addition of 200 µL of buffered [^68^Ga]GaCI_3_ (pH 4.0 and 3.0). The total reaction mixtures (225 µL, pH 4.0 and 3.0) constituting different peptide concentrations (5–303 µM) were heated at 95 °C for 15 min using a heat block. Thereafter, the effect of incubation time (5–20 min) on the complexation of DOTA-AeK and [^68^Ga]GaCI_3_ was reported. Samples of the reaction solution were tested for labeling efficiency and radiochemical purity by performing radio-ITLC and radio-HPLC analysis.

### 3.3. Optimization of a Purification Technique for [68Ga]Ga-DOTA-AeK

A step-by-step radiochemical purification procedure for [^68^Ga]Ga-DOTA-AeK was developed using different solid phase extraction (SPE) cartridges: (a) SepPak C18, (b) SepPak C-8 Plus (light), (c) Oasis hydrophilic-lipophilic balance (HLB) (Waters Corporation, Milford, MA, USA) and (d) Strata X (Phenomenex, Torrance, CA, USA). The essential parameters, including the cartridge binding efficiency, rinse, type of elution solution, and product recovery, were investigated. In brief, the cartridges were pre-conditioned using EtOH, followed by H_2_O. The [^68^Ga]Ga-DOTA-AeK reaction solution was loaded onto the cartridges and rinsed with aqueous-based solutions. The recovery of [^68^Ga]Ga-DOTA-AeK from the cartridge adsorbent was performed using organic solutions. The radioactivity was measured at each step by measuring the loaded cartridges, wash steps, and elution fractions in the dose calibrator. The cartridge loading, rinse/wash, and recovery efficiency was determined as the percentage of radioactivity used out of the total starting radioactivity. Thereafter, the overall purification performance was assessed based on the recovery efficiency (factoring in the loading and elution efficiency) and RCP of the purified product using radio-HPLC.

### 3.4. Radiochemical and Thermodynamical Stability

The stability of [^68^Ga]Ga-DOTA-AeK using samples of the post-purification formulation was investigated for up to 120 min at room temperature by comparing the benchtop condition (pH 4.0) and neutralized product formulation adjusted to pH 7.0 (2 M NaOH) using radio-HPLC, determining the %RCP for each time point.

### 3.5. Log P Determination

The compound hydrophobicity was determined using the octanol–water partition coefficient “shake flask method” described by Lambidis et al. [50]. In brief, 100 µL of purified [^68^Ga]Ga-DOTA-AeK was added to 400 µL of ultrapure water. The aqueous solution was added to an equal volume of 1-octanol (Sigma-Aldrich, Darmstadt, Germany), and the mixture was mechanically shaken for approximately 3 min at room temperature. The sample was centrifuged at 16,211 *g* for 5 min using a Digicen 21 R centrifuge (Orto Alresa, Madrid, Spain). The aqueous and organic phases of the sample were separated, and samples from each phase were analyzed using a dose calibrator. The logarithmic partition coefficient, Log P, was calculated using the following formula:

Log P = Log [(counts in octanol/counts in water)](1)

### 3.6. Proteolytic Stability and Serum Protein Binding of [^68^Ga]Ga-DOTA-AeK

The plasma protein binding filter assay described by Müller et al. [51] was adopted. Before the experiment, Amicon^®^ Ultra-0.5 centrifugal 10 kDa MWCO filter units (Merck, Darmstadt, Germany) were pre-washed with 200 µL of phosphate-buffered saline (PBS) for 20 min at 18,611 *g* using the Microfuge 16 centrifuge (Beckman Coulter Life Sciences, Brea, CA, USA). Subsequently, 800 µL of plasma was spiked with 200 µL of purified [^68^Ga]Ga-DOTA-AeK, vortexed for 3 s, and incubated for 120 min at 37 °C. For radioanalysis, a 100 µL plasma sample was taken at different time intervals (30, 60, and 120 min), diluted (1:1) with PBS, aliquoted into filter units, and centrifuged at 14,800 rpm for 20 min. A similar procedure was carried out in PBS (140 mM NaCl, 10 mM phosphate buffer, and 3 mM KCl, pH 7.4) as a control. After centrifugation, each filter unit was washed by adding 100 µL of PBS (140 mM NaCl, 10 mM phosphate buffer, and 3 mM KCl, at pH 7.4, and centrifuged as described above. The counts of the filtrate fraction (representing unbound compound) and on the filter (protein-bound compound) were measured using a Capintec Captus 4000E well counter (Florham Park, NJ, USA). The percentage of the protein-bound radiotracer was calculated using the following equation:

% Protein bound = [counts in filter ÷ total counts (filtrate fraction+ filter)] × 100(2)

In addition, the filtrated fraction was analyzed via radio-HPLC to evaluate the radiotracer stability in plasma and PBS by determining the %RCP.

### 3.7. Bacterial Cell Uptake of [^68^Ga]Ga-DOTA-AeK

Overnight bacterial cell cultures of *E. coli* or *S. aureus* were grown in Tryptic Soy Broth (TSB) medium at 37 °C. The 6.0 McFarland standard was prepared using DEN-18 McFarland Densitometer (Biosan, Riga, Latvia). For control studies, the same amount of bacterial culture was heat-killed via 30 min incubation at 90 °C. The culture viability was confirmed using agar plating with overnight incubation. All tests were initiated by adding 4.1 ± 1.23 MBq of [^68^Ga]Ga-DOTA-AeK product solution (4.86 ± 1.45 GBq/µmol) to 1.0 mL of the bacterial suspension (1.8 × 10^8^ cells/mL), and mixtures were maintained while shaking at 37 °C. At specific time intervals (10, 60, and 120 min), samples of the suspension were centrifuged at 16,211 *g* for 10 min (Digicen 21R, United Scientific, Goodwood, South Africa), and the supernatant was transferred into a plastic tube. The pellet was washed with 3 mL of PBS (140 mM sodium chloride, 10 mM phosphate buffer, and 3 mM potassium chloride, pH 7.4) and centrifuged at 16,211 *g* for 10 min. For measurement, all volumes of supernatant were pooled into one single liquid fraction. The radioactivity in the supernatant and pellet fraction were measured using an automatic gamma counter (Hidex AMG, LabLogic, Turku, Finland). The percentile of [^68^Ga]Ga-DOTA-AeK cell uptake was calculated as follows:

Uptake (%) = [counts in cell pellet ÷ total counts (cell pellet + supernatants)] × 100(3)

### 3.8. Bacterial Cell Uptake and Incorporation of AeK-NBD

#### 3.8.1. Flow Cytometry

Bacterial cell cultures (either live or heat-killed) of *E. coli* or *S. aureus* (1.0 McFarland standard) were prepared as described above. For bacterial staining, 2.0 μL (100 mM in DMSO) of fluorescent AeK-NBD was added to 198 μL of bacterial culture (7.7 × 10^7^ cells/mL), and cells were counterstained with 2.0 μL (10 μM in DMSO) of membrane-permeable DNA Vybrant DyeCycle (VDC) Ruby dye (Thermo Fisher Scientific, Waltham, MA, USA). All cultures were incubated at 37 °C, and samples were taken at different intervals (10, 30, and 60 min) to perform flow cytometric analysis (Cytoflex, Beckman Coulter Life Sciences, Brea, CA, USA). AeK-NBD was excited with a 488 nm laser, and the emitted fluorescence was collected using a 540/30 nm band pass filter, as carried out previously [25]. The VDC Ruby dye was excited with a 638 nm laser, and the emitted fluorescence was collected using a 712/25 nm band pass filter. No noticeable spillover was observed when VDC Ruby ‘only’ and AeK-NBD ‘only’ controls were used as controls for possible fluorescence spillover. Thus, no compensation was applied when using both dyes. The NBD-AeK median fluorescence intensity (MFI) of VDC Ruby-positive bacteria was reported. Post-acquisition data analysis was performed using Kaluza Analysis Software (version 2.2; Beckman Coulter, Miami, Brea, CA, USA).

#### 3.8.2. Confocal Microscopy

Sample preparation of *E. coli* and *S. aureus* suspensions for confocal fluorescence microscopy imaging involved immediate staining with AeK-NBD and membrane-permeable DNA VDC Ruby dye for 10 min, as described in the previous section. Cells were subsequently fixed in 10% formalin for 15 min, followed by centrifugation at 76,471 *g* for 3 min using a Microfuge 16 centrifuge (Beckman Coulter Life Sciences, Brea, CA, USA), and the cell pellet was washed in PBS. The bacterial suspension was centrifuged, and the cells were deposited in Molwiol/DABCO mounting fluid on a microscope slide and overlaid with a No 1 thickness coverslip. Images were collected on a Zeiss LSM800 confocal microscope (Oberkochen, Germany) using a 63× 1.4 NA oil objective with 2.5× scan zoom and 4× averaging. Furthermore, 488 and 561 excitation wavelengths were used at 0.30% and 1.87% power and 852 V and 845 V, respectively, along with 0.82 and 0.69 AU pinholes.

### 3.9. Exploratory [^68^Ga]Ga-DOTA-AeK-PET/CT Imaging

#### 3.9.1. Animals

All animal handling and experimental procedures were conducted by a licensed veterinary professional and performed with approval (see Institutional Review Board Statement).

Six-to-eight-week-old mixed-gender BALB/c mice (20.0 ± 2.1 g) were utilized. Animals were pre-grouped (two mice/cage) into individually ventilated cages (IVC, Tecniplast, Buguggiate, Italy) and kept under standard housing conditions (22 ± 2 °C, 55 ± 10% humidity, 15 ± 5 Pa, and 12 h light/dark cycles) with food and water being provided ad libitum. Each experimental group consisted of 5 animals.

#### 3.9.2. Establishment of the Murine Infection and Inflammation Animal Model

A thigh muscle mouse model of infection and inflammation was developed for imaging purposes by adopting procedures from a previous study with some modifications [49]. In brief, animals were inoculated intramuscularly (right hind leg) with 1.5 × 10^7^ colony forming units (CFU)/mL of *S. aureus* or *E. coli* suspended in 100 µL of culture media. To induce sterile inflammation, the same mice received 100 µL of a turpentine oil solution intramuscularly into the left hind leg. Infection was allowed to develop for 3–5 days, which is typical for a model of chronic infections, and a health check, including monitoring of the infection and inflammation sites, was conducted. At 3 and 5 days after the inoculations, all animals underwent whole-body [^68^Ga]Ga-DOTA-AeK microPET/CT imaging, and after euthanasia, a complete ex vivo organ tissue biodistribution radioactive assay was performed.

#### 3.9.3. Animal Imaging Procedure

A total volume of 100 µL of [^68^Ga]Ga-DOTA-AeK (7.4 ± 2.0 MBq, 5% EtOH/PBS, pH 7.0) was administered as an intravenous bolus via the tail vein. At the same time, the animals were kept anesthetized, (4% isoflurane in oxygen for induction and 1.5–2.5% for maintenance). Non-invasive, whole-body microPET/CT imaging was performed using a Mediso nanoScan^®^ PET/CT scanner (Budapest, Hungary). A previously described, image acquisition was performed [52]. The PET/CT imaging procedure consisted of static whole-body scans per animal commencing at 60 min post-injection. The imaging protocol included the X-ray topogram (30 s) to ascertain the correct animal placement followed by CT image acquisition (4–5 min) and 20 min long PET image acquisition. Image reconstruction and analysis were performed as previously described [30].

#### 3.9.4. Ex Vivo Biodistribution Studies and Histopathology

After PET/CT imaging, the mice were euthanized via cervical dislocation while still under anesthesia. The different organs (spleen, pancreas, stomach, intestine, kidneys, liver, heart, lung, muscle, and femur) were collected into pre-weighed tubes. The radioactivity of each sample was determined using an automatic gamma counter (Hidex AMG, Turku, Finland). Results were expressed as a percentage of injected dose per gram organ (%ID/g). Subsequently, both muscles on the left and right thighs were fixed in 4% paraformaldehyde and submitted to the University of Pretoria, Department of Paraclinical Science, for hematoxylin and eosin (H&E) and Gram staining. Infection and inflammation tissue analysis was performed by a qualified pathologist.

### 3.10. Statistical Analysis

Unless stated otherwise, the results were analyzed using Microsoft Excel 365 Software (Redmond, WA, USA) and GraphPad Prism 9.5.1 Software (San Diego, CA, USA). Results from experimental repetitions are expressed as mean ± standard deviation (SD). Means were compared using the unpaired two-tailed Student *t*-test, and differences in values were considered statistically significant when *p* < 0.05.

## 4. Conclusions

An initial preclinical investigation of the tripeptide AeK to determine its potential role as a prospective PET imaging agent for the visualization of bacterial infections by the targeting PG salvage pathway was presented. We provided new insights for NBD-AeK analog internalization by Gram-negative bacteria (*E. coli*) and a Gram-positive (*S. aureus*) strain. Motivated by the results, a ^68^Ga radiosynthesis protocol was developed for the DOTA-AeK analog, featuring optimal labeling parameters and a tailored purification procedure that further allowed for initial radiochemical characterization and early bio-pharmacological preclinical investigations. Promising radiochemical and proteolytic compound stability result motivated us to conduct non-invasive small animal PET imaging.

The achieved results from both in vitro and in vivo bacteria uptake studies are sufficient for us to conclude that the overall low uptake rate and cellular turnover, attributed to the large molecular weight, compromise the ability of [^68^Ga]Ga-DOTA-AeK to be qualified as a sensitive and infection-specific radiopharmaceutical. Of interest is that minimal accumulation of [^68^Ga]Ga-DOTA-AeK in response to inflammation was observed; however, whether this might impact the specificity of AeK as a potential radiotracer in infection imaging remains unclear.

While the results of this study prove that AeK is a PG biosynthesis-targeting molecule (even when conjugated to NBD), intracellular or membrane incorporation of [^68^Ga]Ga-DOTA-AeK will be a prerequisite for thriving dedicated bacteria-specific imaging. Plausibly, ^68^Ga-DOTA functionalization of AeK (i.e., a necessary replacement of the NBD entity) made the resulting molecule lose valuable functionality for the PG recycling pathway by hindering active membrane transportation. Addressing such a shortcoming, the approach for future studies will focus on preserving the targeting moiety of AeK tripeptide with minimal structural alterations, which can be achieved using alternative direct radiolabeling strategies without the need for radioisotope chelation (e.g., ^11^C and ^18^F, or ^131/124^I). This will include exploration into the diverse chemical structures of AeK tripeptide and subsequent structure–activity relationship investigations for the optimization and improvement of biodistribution and specificity.

## Figures and Tables

**Table 1:** Influence of reducing washing agent volume on purification performance of SepPak C18 light (in-tandem alignment connected back-to-back).

Treatment (Wash & Rinse)	Activity Retention (%LA)	Activity Retention (%LA) Post-Wash	Recovery Efficiency (%RA)	RCP (%)
1 mL PBS (n = 9)	99.7 ± 0.2	85.8 ± 6.9	60.7 ± 12.7	100
0.4 mL PBS (n = 6)	99.8 ± 0.1	94.9 ± 6.2	83.5 ± 7.6	100

The SepPak C18 tandem setting was pre-conditioned with 10 mL of EtOH and 10 mL of H_2_O. Product activity was recovered from the cartridge via slow elution with 1 mL of 5% *v*/*v* EtOH (aqua) solution. Before purification, the RCP was determined via HPLC analysis of a crude reaction solution of ≥95%. %LA = percentage of the total loaded radioactivity from the starting radioactivity, with %LA measured (MBq) on the cartridges after the loading step pre- and post-wash. %RA = percentage of the total recovered radioactivity from the starting radioactivity with RA measured (MBq) after the activity elution (recovery) step.

**Figure A6:**
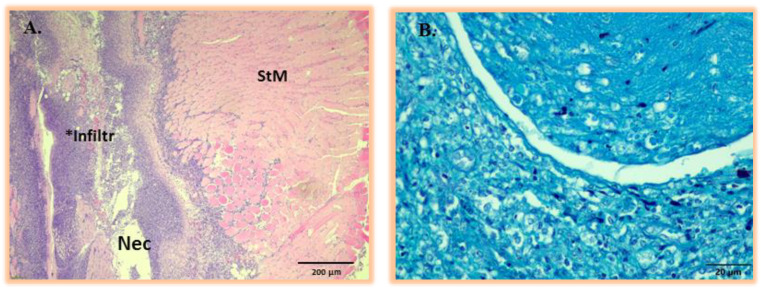
(**A**) Representative histopathology analysis of thigh muscular tissue showing inflammatory response to sterile turpentine oil injection at Day 3 and 5 post-inoculation. All the turpentine-induced tissue lesions are identical, presenting severe necrotic tissue areas (Nec) and the infiltration of neutrophils and macrophages (area marked Infiltr *) within adipose and striated muscle (StM). (**B**) Corresponding Gram-stained sections of thigh muscular tissue at Day 3 and 5 post-inoculation with turpentine oil showing the absence of bacterial strains.

## Data Availability

Data are contained within the article and Appendix A.

## Abbreviations

AeK	L-Ala-γ-D-Glu-L-Lys
CFU	Colony Forming Units
CT	Computed Tomography
DOTA	4,7,10 tetraaza-cyclododecane-N,N′,N″,N‴-tetraacetic acid
*E. coli*	*Escherichia coli*
HLB	Hydrophilic–lipophilic balance
HPLC	High-performance liquid chromatography
%ID/g	Percentage of injected dose per gram organ
ITLC	Instant thin-layer chromatography
IVC	Individually ventilated cages
MFI	Median fluorescence intensity
MRI	Magnetic resonance imaging
NBD	N-7-nitro-2,1,3-benzoxadiazol-4-yl
Opp	Oligopeptide permease
PBS	Phosphate-buffered saline
PET	Positron emission tomography
PG	Peptidoglycan
R*f*	Retention factors
R*s*	Resolution
RCP	Radiochemical purity
*S. aureus*	*Staphylococcus aureus*
SD	Standard deviation
SPE	Solid-phase extraction
TSB	Tryptic soy broth
UDP-MurNAc	Uridine diphosphate -N-acetylmuramic acid
VDC	Vybrant DyeCycle

## Appendix A. Supplementary Data

### Appendix A.1. Material

#### General Information

All chemicals and solvents were purchased from Merck (Darmstadt, Germany), Thermo Fisher Scientific (Waltham, MA, USA), Sigma-Aldrich (St. Louis, MO USA), and Anatech Analytical Technology (Gauteng, South Africa). The AeK-NBD (509.48 g/mol) and DOTA-AeK (732.79 g/mol) peptides were purchased from GLBiochem (Shanghai, China). All the reagents used were prepared using ultra-purified water produced in-house by a Simplicity 185 Millipore system (Merck, Darmstadt, Germany). ^68^Ga-activity was produced by a SnO_2_ matrix-based ^68^Ge/^68^Ga generator (iThemba Labs, Cape Town, South Africa) and eluted in 0.6 M hydrochloric acid (HCI). The eluted radioactivity was routinely measured using a Capintec CRC-25R dose calibrator (Florham Park, NJ, USA); eluate acidity was determined using MQuant pH indicator strips (Merck, Darmstadt, Germany). The purity of DOTA-AEK was determined via a Waters Acquity LC/MS system equipped with a Waters XBridge BEH C18 column (3.5 µm, 100 mm × 3 mm, 130 Å) at a flow rate of 5 mL/min using a gradient of Acetonitrile 0.1% formic acid (5–95% over 10 min) and Water 0.1% formic acid. Instant thin layer chromatography (ITLC) using TLC-Silical gel (TLC-SG)-impregnated paper (Agilent Technologies, Santa Clara, CA, USA) as the stationary phase was conducted for the analysis of [68Ga]Ga-DOTA-AeK using a Veenstra VCS-201 multi-channel chromatogram (Veenstra Instruments, Friesland, The Netherlands). The bacterial strains *Escherichia coli* (*E. coli*, ATCC 29213) and *Staphylococcus aureus* (*S. aureus*, ATCC 29213) were provided by the Department of Medical Microbiology, University of Pretoria, South Africa.

### Appendix A.2. Results

#### Appendix A.2.1. Testing of Radioanalytical Methods for [^68^Ga]Ga-DOTA-AeK Quality Control

##### HPLC and LC/MS

The chemical purity of DOTA-AeK was determined via C18-reverse-phase HPLC. LC/MS analysis of DOTA-AEK confirmed that the compound was pure and demonstrated the correct mass (calculated 732.79 g/mol) with the presence of the following peaks: 733.4 ([M+H]^+^), 734.4 ([M+H]^2+^), 755.40 ([M+H+Na]^+^), 756.35 ([M+H+Na]^2+^), 367.39 (½[M+H]^1+^), and 368.13 (½[M+H]^2^) (Figure A1). The chromatogram showed a dominant peak at a retention time of 5.2 min, which was not detected in the run with pure water (Figure A2A)

The radio-HPLC analysis of [^68^Ga]Ga-DOTA-AeK is shown in Figure A2B, indicating a retention time of 5.9 min. This peak is clearly separated from the retentions of ^68^Ga species eluting at ≤1.5 min. The assay provided an appropriate resolution, >1.5 (i.e., baseline peak separation), to analyze the radiochemical purity and was able to observe plausible radiolabeled by-products.

##### ITLC

Radio-ITLC methods A and B were tested to study the fit-for-purpose aspect of the method and determine the %RCP and %RCY (Figure A3 and Figure A4). Both samples demonstrate the confirmed [^68^Ga]Ga-DOTA-AeK product acquired during radio-HPLC analysis. Results for R*f* and R*s* calculations are displayed in Table A1.

**Table A1:** ITLC retention factors and method resolution for [^68^Ga]Ga-DOTA-AeK.

Mobile Phase A			Mobile Phase B	
[^68^Ga]Ga-DOTA-AeK		[^68^Ga]GaCl_3_ (Ionic + Colloidal)	[^68^Ga]Ga-DOTA-AeK	[^68^Ga]GaCl_3_ (Ionic + Colloidal)
R*f*	0.2	0.7	0.7	0.1
R*s*	1.6		1.1	

Mobile phase A: 0.1 M citric acid (pH 5.0); mobile phase B: 1 M ammonium acetate: methanol (1:1). R*f*: retention factors; R*s*: resolution.

#### Appendix A.2.2. Development of a Purification Method of [^68^Ga]Ga-DOTA-AeK

**Table A2:** Summary of results comparing different purification strategies.

Cartridge-Absorbent	Conditioning (Volume/Agent)	Activity Retention (%_LA_)	Wash and Rinse (Volume/Agent)	Activity Retention (%_LA_) Post-Wash	Activity Elution (Volume/Agent)	Recovery Efficiency (%_RA_)	%RCP
(A) C-18 145 mg	4 mL EtOH + 2 mL H_2_O	72.8	1 mL H_2_O	-	1 mL 50% *v*/*v* EtOH *	37.3	39.9
	4 mL EtOH + 2 mL H_2_O	92.6	1 mL Saline	60	1 mL 50% *v*/*v* EtOH **	46.2	11.8
	4 mL EtOH + 2 mL H_2_O	89.2	1 mL PBS	55.7	1 mL 50% *v*/*v* EtOH *	41.6	11.9
(B) C-8 145 mg	3 mL EtOH + 3 mL H_2_O	83.8	1 mL Saline	32.5	1 mL 100% *v*/*v* EtOH	19.4	12.6
(C) HBL 200 mg	1 mL EtOH + 1 mL H_2_O	42.4	1 mL H_2_O	-	1 mL 100% *v*/*v* EtOH	5.5	11.2
(D) Strata X 100 mg	4 mL EtOH + 2 mL H_2_O	63.6	1 mL H_2_O	7.5	1 mL 100% *v*/*v* EtOH	6.1	19.7
(A–A) C-18 290 mg	10 mL EtOH + 10 mL H_2_O	98.4	1 mL 50% EtOH *	35.2	1 mL50% *v*/*v* EtOH *	29.1	15.3
	10 mL EtOH + 10 mL H_2_O (n = 9)	99.7 ± 0.2	1 mL PBS	85.8 ± 6.9	1 mL 5% *v*/*v* EtOH **	60.7 ± 12.7	100
	10 mL EtOH + 10 mL H_2_O	99.4	0.4 mL H_2_O	93.8	1 mL 10% *v*/*v* EtOH *	78.3	39.9
	10 mL EtOH + 10 mL H_2_O (n = 6)	99.8 ± 0.1	0.4 mL PBS	94.9 ± 6.2	1 mL 5% *v*/*v* EtOH **	83.5 ± 7.6	100

All listed purifications were performed once with the known %RCP determined via HPLC analysis from crude reaction solution > 95%. %_LA_ = the percentage of the total loaded radioactivity from the starting radioactivity with the LA measure (MBq) on the cartridges after the loading step pre- and post-wash. %_EA_ = the percentage of the total recovered radioactivity from the loaded cartridges’ radioactivity with EA measured (MBq) after activity elution from the cartridge post-wash. %_RA_ = the percentage of the total retained radioactivity on the cartridges from the starting radioactivity with RA measured (MBq) after the activity elution (recovery) step. A–A= tandem setup where cartridges were connected back-to-back. (*) in water; (**) PBS (pH 7.4).

#### Appendix A.2.4. [^68^Ga]Ga-DOTA-AeK- Ex Vivo Biodistribution

**Table A3:** Ex vivo organ biodistribution (%ID/g) of [^68^Ga]Ga-DOTA-AeK in a dual-mice model of infection (*E. coli* or *S. aureus)* and inflammation at Day 3 and 5 post-inoculation.

Post-Inoculation	*E. coli*			*S. aureus*		
	Day 3 (n = 4)	Day 5 (n = 4)	* *p*-Value	Day 3 (n = 5)	Day 5 (n = 3)	* *p*-Value
Brain	0.1 ± 0.0	0.2 ± 0.2	0.344	0.1 ± 0.0	0.2 ± 0.0	0.030
Thyroid	1.0 ± 0.7	0.7 ± 0.3	0.497	0.9 ± 0.5	1.9 ± 0.3	0.020
Heart	0.8 ± 0.4	1.6 ± 1.3	0.299	1.2 ± 0.3	3.7 ± 0.5	0.000
Lungs	1.0 ± 0.1	2.0 ± 0.4	0.003	1.8 ± 0.5	3.7 ± 0.5	0.002
Liver	1.1 ± 0.2	1.2 ± 0.4	0.631	1.5 ± 0.5	3.6 ± 1.5	0.024
Spleen	0.7 ± 0.3	0.7 ± 0.1	0.986	1.4 ± 1.6	1.6 ± 0.6	0.801
Pancreas	0.6 ± 0.3	0.8 ± 0.4	0.450	0.7 ± 0.3	2.3 ± 0.2	0.000
Stomach	0.3 ± 0.1	1.3 ± 1.8	0.295	0.7 ± 0.5	1.1 ± 0.4	0.347
Intestines	0.4 ± 0.2	1.1 ± 1.2	0.356	1.2 ± 1.9	1.8 ± 0.8	0.653
Kidneys	3.8 ± 1.6	2.9 ± 0.7	0.374	5.6 ± 3.4	7.0 ± 0.1	0.512
Adipose	0.7 ± 0.1	0.8 ± 0.7	0.459	1.0 ± 0.4	1.9 ± 0.3	0.010
Femur	1.4 ± 1.0	0.9 ± 0.4	0.453	1.2 ± 1.3	2.0 ± 0.2	0.335
Muscle	0.7 ± 0.4	0.6 ± 0.2	0.632	0.4 ± 0.4	1.1 ± 0.5	0.041
Inflammation	0.9 ± 0.0	0.9 ± 0.1	0.987	1.1 ± 0.5	2.6 ± 0.6	0.010
Infection	0.4 ± 0.1	0.4 ± 0.0	0.398	0.8 ± 0.6	0.9 ± 0.3	0.781
Inflammation/muscle	2.0 ± 1.6	1.8 ± 0.8	0.492	7.1 ± 9.9	2.1 ± 0.3	0.064
Infection/muscle	0.8 ± 0.5	0.8 ± 0.3	0.771	3.0 ± 0.2	0.8 ± 0.1	0.559

* Unpaired *t*-tests were performed for comparison; *p* < 0.05 was considered statistically significant.

#### Appendix A.2.5. Histopathology

The H&E staining of both *E. coli* and *S. aureus* infection sites revealed variably mild to moderate inflammatory foci consisting of the infiltration of macrophages, neutrophils, and lymphocytes (granulomatous inflammation) (Figure A5A,B). Despite the inflammatory markers observed for both *E. coli* and *S. aureus* infection, the presence of bacteria intralesionally could not be confirmed for both *E. coli* and *S. aureus* infection via H&E and Gram staining (Figure A5C,D). As previously reported, this might have been due to the heterogeneous low-density bacterial distribution and phagocytosis of bacteria by macrophages [53]. As a study limitation, bacterial culturing, which is a more sensitive technique for verifying the presence of active infection, was not carried out [54].

The tissue responses to turpentine-induced severe intramuscular inflammation were mainly characterized by necrosis, edema, and neovascularization, and were similar in all mice at Day 3 and Day 5 post-induction. As expected, no positive bacteria staining was confirmed in the inflammation area (Figure A6A,B).
